# Prognostic microRNAs modulate the RHO adhesion pathway: A potential therapeutic target in undifferentiated pleomorphic sarcomas

**DOI:** 10.18632/oncotarget.3926

**Published:** 2015-04-23

**Authors:** Philip Wong, Angela Hui, Jie Su, Shijun Yue, Benjamin Haibe-Kains, Nalan Gokgoz, Wei Xu, Jeff Bruce, Justin Williams, Charles Catton, Jay S. Wunder, Irene L. Andrulis, Rebecca Gladdy, Brendan Dickson, Brian O'Sullivan, Fei-Fei Liu

**Affiliations:** ^1^ Radiation Medicine Program, Princess Margaret Cancer Centre, University Health Network, Toronto, ON, Canada; ^2^ Department of Radiation Oncology, University of Toronto, Toronto, ON, Canada; ^3^ Département of Radiation Oncology, Centre Hospitalier de L'Université de Montréal, Montréal, QC, Canada; ^4^ Research Institute, Princess Margaret Cancer Centre, University Health Network, Toronto, ON, Canada; ^5^ Department of Biostatistics, Princess Margaret Cancer Centre, Toronto, ON, Canada; ^6^ The Lunenfeld-Tanenbaum Research Institute, Mount Sinai Hospital, Toronto, ON, Canada; ^7^ University Musculoskeletal Oncology Unit, Mount Sinai Hospital, Toronto, ON, Canada; ^8^ Department of Surgery, University of Toronto, Toronto, ON, Canada; ^9^ Department of Molecular Genetics, University of Toronto, Toronto, ON, Canada; ^10^ Department of Laboratory Medicine and Pathobiology, University of Toronto, Toronto, ON, Canada; ^11^ Department of Pathology, Mount Sinai Hospital, University of Toronto, Toronto, ON, Canada; ^12^ Department of Medical Biophysics, University of Toronto, Toronto, ON, Canada

**Keywords:** biomarker, sarcoma, microRNA, prognostic, metastasis

## Abstract

A common and aggressive subtype of soft-tissue sarcoma, undifferentiated pleomorphic sarcoma (UPS) was examined to determine the role of micro-RNAs (miRNAs) in modulating distant metastasis. Following histopathologic review, 110 fresh frozen clinically annotated UPS samples were divided into two independent cohorts for Training (42 patients), and Validation (68 patients) analyses. Global miRNA profiling on the Training Set and functional analysis *in vitro* suggested that miRNA-138 and its downstream RHO-ROCK cell adhesion pathway was a convergent target of miRNAs associated with the development of metastasis. A six-miRNA signature set prognostic of distant metastasis-free survival (DMFS) was developed from Training Set miRNA expression values. Using the six-miRNA signature, patients were successfully categorized into high- and low-risk groups for DMFS in an independent Validation Set, with a hazard ratio (HR) of 2.25 (*p* = 0.048). After adjusting for other known prognostic variables such as age, gender, tumor grade, size, depth, and treatment with radiotherapy, the six-miRNA signature retained prognostic value with a HR of 3.46 (*p* < 0.001). A prognostic miRNA biomarker for clinical validation was thus identified along with a functional pathway that modulates UPS metastatic phenotype.

## INTRODUCTION

Soft tissue sarcomas (STS) are a conglomerate of mesenchymal tumors, which represent 1% of all human malignancies [1]. One of the most common STS subtypes is undifferentiated pleomorphic sarcoma (UPS), which is amongst the most aggressive STS with a high propensity for distant metastasis (DM), resulting in dismal five-year overall survival (OS) rates ranging from 30-50% [2, 3]. Clinical prognostic determinants in STS [4] have not been helpful in identifying patients who might benefit from systemic chemotherapy [5]. Thus, there remains a significant need to develop novel biomarkers, which will provide both insights into biology, as well as facilitating individualization of cancer therapy.

MicroRNAs (miRNAs) are small non-coding RNA molecules of ~22-nucleotides that form one of the largest classes of gene regulators since they are predicted to target more than 30% of mammalian mRNAs through translational repression or degradation [6]. Innumerable studies have characterized miRNA dysregulation in human malignancies [7, 8]. To date, three studies have described miRNA expression patterns for sarcomas, demonstrating that such profiles can distinguish some STS histologies from others [9-11]. Hisaoka *et al* further reported that modulation of miR-let-7e and miR-99b reduced synovial sarcoma cell proliferation, suggesting a potential role for these miRNAs in STS [10]. To date however, there have been no reports of miRNA profiling of STS in relation to clinical outcome.

In this study, we hypothesized that miRNAs mediate the metastatic ability of UPS. Expression profiling of miRNAs from 42 primary UPS identified 40 miRNAs associated with distant metastasis-free survival (DMFS). Functional and pathway evaluations suggested that miR-138 and its downstream RHOA/C (Ras homolog gene family, member A/C)~ROCK1/2 (Rho kinase 1/2)~LIMK1/2 (LIM kinase 1/2) cell adhesion pathway appeared to be a convergent target of DMFS-associated miRNAs. A prognostic signature based on the expression level of six miRNAs was developed from the Training Set, and then validated using an independent cohort of UPS samples prospectively linked to clinical outcome.

## RESULTS

### Identifying prognostic miRNAs that modulated UPS biology

Global miRNA profiling of the Training Set demonstrated that 166 (43.9%) of the miRNAs were significantly under-expressed in UPS primaries, compared to normal tissues (*p* < 0.0001); no miRNAs were significantly over-expressed in UPS (Supplementary Figure 1).

The expression level of 40 miRNAs was significantly associated with DMFS (Supplementary Table 1); many of these genes had been previously associated with increased risk of developing metastasis in other malignancies. Pathway analysis using DIANA miRPath V2.0 [12] suggested that in addition to the MAPK pathway, the Focal Adhesion cascade was targeted by 31 of these 40 miRNAs associated with DMFS.

### MiRNA-138 promoted invasion of sarcoma cells

**Figure 1:**
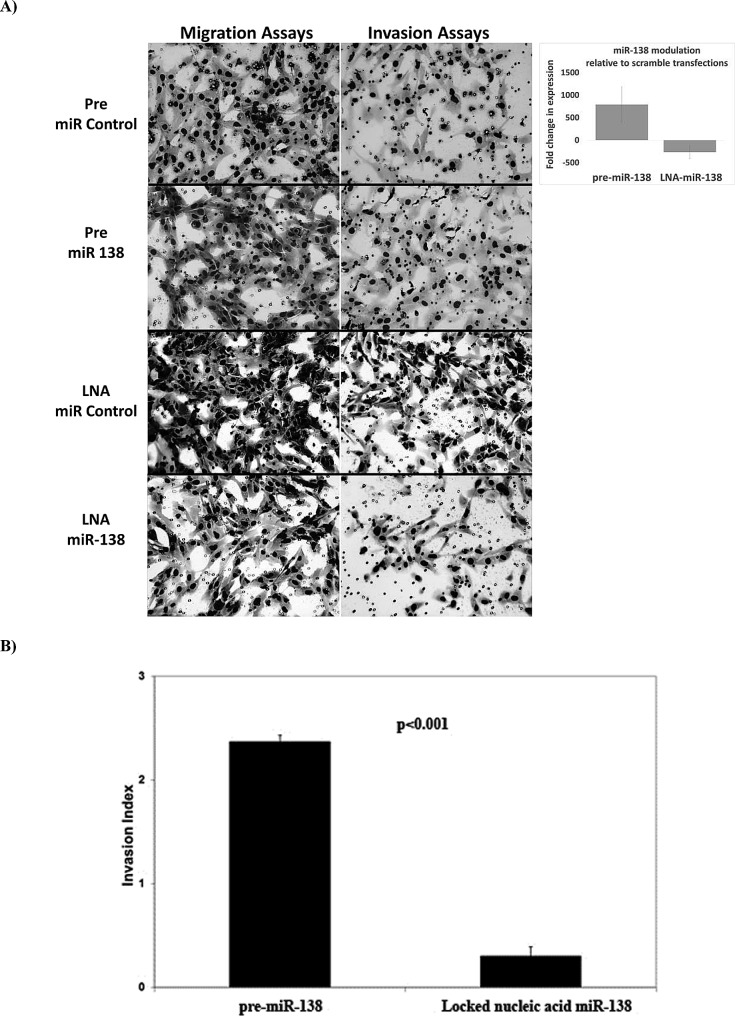
Cell morphology and invasion phenotype following miR-138 modulation Images of membrane areas populated by cells transfected with control (pre-miR-scrambled and LNA-scrambled), pre-miR-138 and LNA-miR-138 are shown: **A.** to illustrate cellular morphology. Cells transfected with pre-miR-138 demonstrated a loss of spindle shape, while control and LNA-miR-138 transfected cells remained spindle-like. The levels of miR-138 expression following LNA-miR-138 and pre-miR-138 transfections (in relation to cells transfected with scrambled controls) are illustrated in the upper right panel. **B.** The invasion indices, which represented the invasive ability of cells over their migration ability, were calculated as (Invasion/Migration of Test Cell)/( Invasion/Migration of Control Cell) for each condition. The invasion indices shown in **B.** are in relation to the invasiveness of cells transfected with control conditions (pre-miR or LNA-scrambled), which would have been assigned an invasive index of 1. Data are presented as the mean + standard error of the mean.

To explore whether miRNAs modulated metastasis, we probed the biological effects of miRNAs that were related to DMFS. Due to the number of miRNAs, screening assays were focused on the top 9 miRNAs (miR-15, 21, 128, 130a, 138, 139-5p, 224, 375 and 491-5p) most significantly correlated with DMFS, or known to modulate cellular adhesion and metastasis in other cancers. Initial screening using migration and invasion assays suggested that knock-down of miR-128, miR-130a, miR-138 and miR-224 reduced migration and invasion of STS117 cells; hence these miRNAs were further evaluated for clonogenic survival following miRNA modulation. The combined result of the assays indicated that miRNA-138 and miRNA-224 were the best candidates to interrogate further as these two miRNAs were individually associated with both DMFS and DFS (Supplementary Figure 2); moreover, *in vitro* experiments demonstrated that increased expression of miRNA-138 and -224 promoted cell invasion; conversely, their knock-down decreased invasion (Figure 1). However, while knock-down of miRNA-138 had no effect on clonogenic survival (Supplementary Figure 3), or cell cycle (data not shown); miRNA-224 was cytotoxic (Supplementary Figure 3). Of note, prior to miRNA manipulation, STS 117 ΔCt levels of miR-138 and miR-224 were 5.4 higher (42-fold) and 3.5 higher (11-fold) respectively, than the average ΔCt of primary UPS from the Training Set. The levels of miRNA modulation were verified following transfections (Figure 1A; right hand panel).

To pursue potential downstream mRNA targets and pathways of miR-138 and miR-224, global mRNA expression analysis was performed on STS117 cells transfected with LNA-miR-138 and LNA-miR-224, which reduced miRNA-138 and miRNA-224 levels by a mean of 266- and 1265-fold, respectively. This list of genes was combined with already-described targets of miRNA-138 and miRNA-224 such as *RHOC* and *ROCK2* [13, 14] to identify potential pathways that could promote invasion in UPS. Pathway analysis using DAVID [15] and g-profiler [16] suggested that miRNA-138 and miRNA-224 target genes were associated with adhesion pathways involving *RHOC* and *ROCK2*. QRT-PCR experiments confirmed previous reports [13, 14] indicating that overexpression of miR-138 reduced the mean mRNA levels of *RHOC* and *ROCK2* by 3.7 and 2.8-fold, respectively (Supplementary Figure 4A). Western blot analysis demonstrated the anticipated reduction of RHOC protein level post-miR-138 transfection (Supplementary Figure 4B). However, ROCK1/2 levels and LIMK1/2 phosphorylation increased post-transfection, likely secondary to the dis-inhibition of RHOA by the reduced level of RHOC (Supplementary Figure 4B and 4C).

Given the close association between the functions of RHOA and RHOC in activating downstream Rho-associated kinases [17], the potential relevance of *RHOA and RHOC* in UPS metastasis was evaluated in 28 samples from the Validation Set (14 patients who developed metastasis, and 14 patients without metastasis), plus 10 lung metastases obtained from metastatectomies (Supplementary Figure 4D). UPS patients who subsequently developed metastases had significantly lower *RHOA* (no correlation with RHOC) expression in their primary tumors, compared to those who never developed metastases (*p* = 0.006; Supplementary Figure 4D). Moreover, *RHOA* transcript expression level was even further reduced in the lung metastasis specimens (*p* < 0.001; Supplementary Figure 4D).

**Figure 2:**
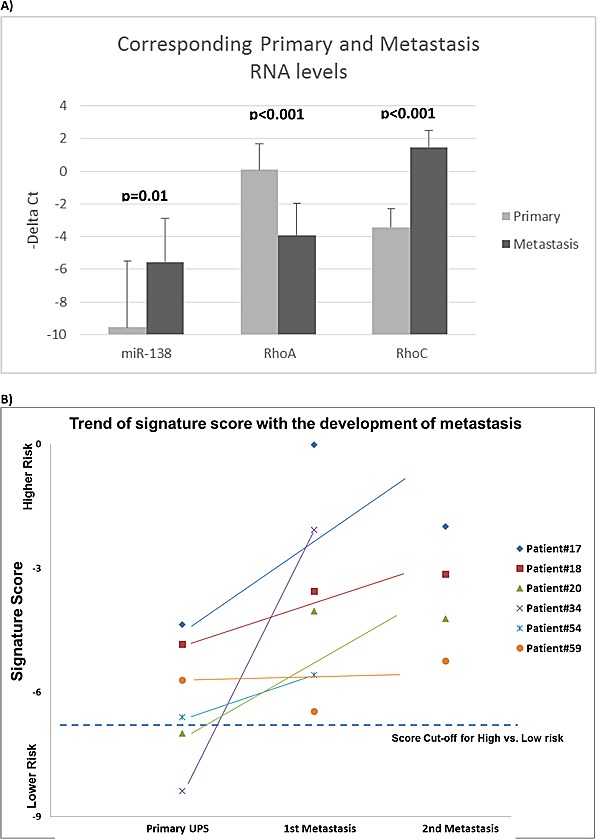
miRNA signature risk score and Rho expressions in primary and metastatic sarcomas Evaluation of the 6-miRNA prognostic signature score **A.** in 6 primary UPS samples from the Validation Set and their corresponding surgically resected lung metastases. Prognostic scores of the metastases suggested that they were all “High-Risk”. Nine of the 10 lung metastases had higher risk scores than their corresponding primaries. The mRNA expression levels of miR-138, RhoC and RhoA were measured in the metastases and their corresponding primary tumors **B.** Data are presented as –ΔCt (−Delta Ct), where higher values represent higher expression. MiR-138 expression was significantly (*p* = 0.01) higher in metastases than in their originating primaries. RhoA expression was reduced in metastases (*p* < 0.001); RhoC expression was increased in metastases (*p* < 0.001).

The RNA levels between the metastases and their corresponding primary tumors were measured. The level of miR-138 in metastasis was significantly higher than their corresponding primaries (*p* = 0.01) (Figure 2A). The level of RhoA mRNA was significantly lower in metastases than in their corresponding primaries (*p* < 0.001); however, RhoC mRNA expression was higher in metastases compared to their corresponding primaries (*p* < 0.001). Concordantly, the expressions of RhoA and RhoC were inversely related (ANOVA *p* < 0.001). There was no significant correlation between the level of miR-138 with RhoA or RhoC expression.

### MiRNA signature as potential prognostic biomarker in the clinic

**Figure 3:**
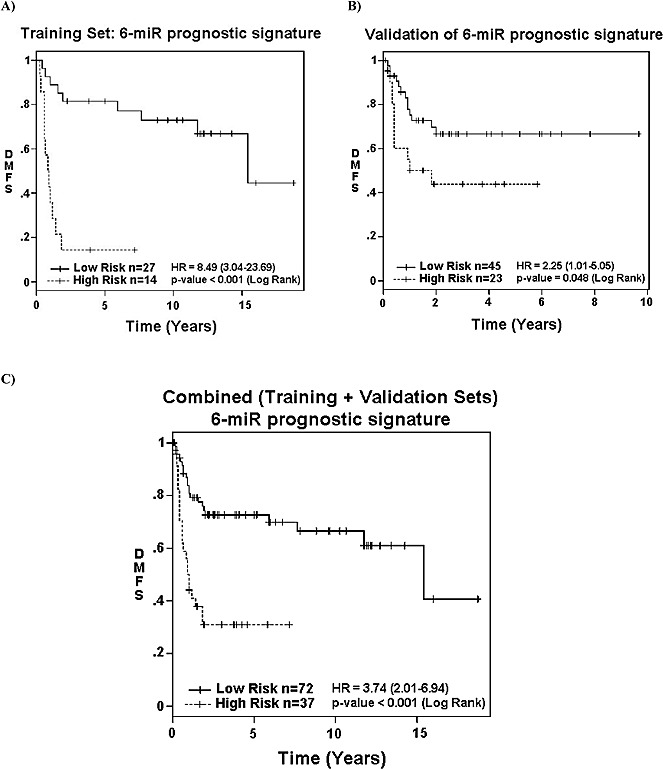
Association of 6-miRNA signature with survival Association of the 6-miRNA prognostic signature with distant metastasis free survival (DMFS) in: **A.** the Training-Set (*p* < 0.001); **B.** Validation-Set (*p* = 0.048); and **C.** combined datasets (*p* < 0.001) based on their “High” *vs*. “Low” risk categorization.

As many of the DMFS associated miRNAs directly or indirectly modulated different members of the RHO and ROCK family, we sought to develop a miRNA prognostic signature based on univariate and multivariate modeling of the miRNA expressions from the Training Set. A prognostic signature score for DMFS consisting of six miRNAs and their regression coefficients was developed: Risk Score = −0.15*miR-132 expression −0.299*miR-138 expression −0.217*miR-143 expression +0.427*miR-221 expression −0.334*miR-224 expression −0.35*miR-491-5p expression. Accordingly, patients were then dichotomized into “Low-risk” (score < −6.7) *vs*. “High-risk” (score > −6.7) categories, with a HR for DMFS of 8.49 (*p* < 0.001) (Figure 3A).

To validate this candidate signature, the expression level of these six miRNAs was assessed using single-well qRT-PCR from the Validation Set (*n* = 68), and inserted into the above formula. By design, patients from the Validation Set were treated more recently than those in the Training Set; hence the Validation Set had shorter follow-up time (*p* < 0.001; Table 1). The Validation Set also comprised of slightly older patients (*p* = 0.028), with larger tumors (*p* < 0.001). Despite the aforementioned differences, the 5-year OS for the two cohorts were similar (54% *vs.* 58%; *p* = 0.688) (Supplementary Figure 5).

**Table 1:** Characteristics of the patients in each sarcoma cohort Patient, tumor and treatment characteristics of the UPS “Training Set” and “Validation Set”

Factors	Training Set N = 42	Validation Set N = 68	*P*-value
**Year of diagnosis**	1988-1999	2000-2010	
**Gender** **M:F**	23:18	41:27	0.67
**Median age in years (range)**	64 (35-95)	68 (32-90)	0.028
**Median size in cm (range)**	6.25 (1.3-28)	11.5 (2.2-28)	0.0004
**Grade** **2** **3**	11 (26%) 31 (74%)	14 (21%) 54 (79%)	0.73
**Stage** **I/II** **III**	17 (40%) 25 (60%)	26 (38%) 42 (62%)	0.82
**Depth** **Deep** ** Superficial**	88% 12%	78% 22%	0.18
**Adjuvant Chemo** **No** **Yes**	41 (98%) 1 (2%)	67 (98%) 1 (2%)	0.72
**Adjuvant RT** **No** **Yes**	12 (29%) 30 (71%)	22 (32%) 46 (68%)	0.54
**Median follow-up in months (range)**	123 (18-225)	28.5 (0-116)	<0.0001
**Local recurrence**	9 (21%)	9 (13%)	
**Metastasis**	16 (38%)	17 (25%)	
**Deaths**	18 (43%)	18 (26%)	
**Disease free survival events**	25 (60%)	27 (40%)	
**Metastasis or deaths**	21 (50%)	24 (35%)	

Notes: the event rates were higher in the training than the validation sets because the training set had a much longer follow-up time.

In the Validation Set, “High-risk” patients were 2.25 times more likely to develop metastasis than “Low-risk” patients on univariate analysis (Figure 3B; *p* = 0.048). After adjusting for known prognostics factors, the signature retained the ability to discriminate patients at “High” *vs*. “Low” risk of developing DM (HR 3.46; *p* < 0.001) in the combined groups of patients (Training Set plus Validation Set) (Figure 3C; Table 2). The only other variable that retained prognostic value for DMFS was tumor depth, but with a lower HR of 0.28 (*p* = 0.039; Table 2).

**Table 2:** The prognostic value of the 6-miR signature in Undifferentiated Pleomorphic Sarcoma The prognostic value of the 6-miR signature was determined using univariate and multivariate analyses of the 6-miR signature using the primary endpoint, distant metastasis free survival (DMFS) and other endpoints (distant control (DC), disease free survival (DFS) and overall survival (OS)) from the combined UPS cohorts of “Training Set” and “Validation Set”. The multivariate analyses of the 6-miR signature score for its ability to predict the primary endpoint, DMFS demonstrated that beside the 6-miR signature, the only other clinical factor that was prognostic of DMFS was tumor depth. All multivariate analyses included the following parameters: 6-miR signature risk, Gender, Age at diagnosis, Tumor size, Tumor Grade, Tumor Depth and use of Radiotherapy

Univariate analyses*		DMFS	DC	DFS	OS
		Hazard ratio (95% CI)			
6-miR signature risk	High vs. Low	3.74 (2.01-6.94)	4.24 (2.10-8.58)	2.97 (1.67-5.27)	3.33 (1.68-6.62)
**Multivariate analyses**					
6-miR signature risk	High vs. Low	**3.46** **(1.84-6.51)** **p=0.0001**	3.65 (1.78-7.51) **p<0.001**	2.91 (1.62-5.25) **p<0.001**	4.06 (1.97-8.38) **p<0.001**
Gender	F vs. M	0.56 (0.3-1.05) p=0.072			
Age at diagnosis	Continuous	1.02 (1-1.04) p=0.1			
Tumor Size	Continuous	1.04 (0.99-1.09) p=0.11			
Tumor Grade	2 vs. 3	0.7 (0.33-1.49) p=0.36			
Tumor Depth	Sup. vs. Deep	0.28 (0.08-0.93) p=**0.039**			
Radiotherapy	No vs. Yes	1.11 (0.55-2.23) p=0.77			

* Univariate correlations in table are significant at p<0.001

In addition, the expression levels of these six miRNAs were measured in 10 resected UPS pulmonary metastases, derived from six patients. As shown in Figure 3, the signature score for all 10 metastases were in the “High-risk” category. Furthermore, with the exception of 2 samples, all other original UPS samples had the “High-risk” signature score (Figure 2B). Specifically, the expression of miR-138 and miR-143 were significantly (*p* = 0.040 and < 0.001 respectively) over-expressed (20.5- and 10.2-fold, respectively) in the metastases as compared to their original primary UPS specimens.

### *In silico* analysis of the potential prognostic value of the six miRNA signature in breast cancer

Application of the six-miRNA-signature was explored in the TCGA BRCA dataset [18] (*n* = 762) motivated by: a) similarity between the observed morphological changes in miR-138 modulated UPS cells with previously described changes in breast cancer cells following *RHOA/C* modulation [17]; and b) prior success in cross-validating the “cinsarc” sarcoma-derived mRNA signature with breast cancer [19]. After adjusting for age and stage, the six-miRNA signature was observed to be prognostic for OS in the BRCA dataset (HR = 1.63; *p* = 0.039) (Table 3). We then asked whether the expression of other genes within the Rho family and ROCK-LIMK pathway (Supplementary Figure 4C) might also correlate with clinical outcome. Indeed, on univariate analysis, six variables (Stage, *RHOA, RHOBTB2, RHOC, RHOG* and *SSH1*) were significantly associated with OS (*p* < 0.05; Supplementary Table 2); furthermore, the expression level of six specific genes in the RHO-ROCK-LIMK pathway (*RHOA, RHOBTB2, RHOC, RHOG, ROCK2, SSH3*) also interacted significantly (*p* < 0.05) with the six-miRNA signature scores, strongly suggesting a potential corroborative nature between these miRNAs and the RHO-ROCK-LIMK pathway. The association between *RHOA* with breast cancer DMFS was further examined using the “Combined breast dataset” (*n* = 1056). On univariate analysis, low *RHOA* expression was again significantly associated with shorter DMFS (Supplementary Figure 6; *p* = 0.014); multivariate analysis could not be performed due to lack of additional clinical data.

**Table 3:** MiRNA signature score is associated with survival of breast cancer patients Multivariate Cox proportional hazard regression analysis for overall survival (OS) of the 6-miR signature in breast cancers (BRCA) from the TCGA database. Signature score and patient age were dichotomized using the median values. Patients with no-follow up (n = 54) were excluded from analysis. After adjusting for age and stage, the six-miRNA signature was observed to be prognostic for OS in the BRCA dataset

Factors	Hazard Ratio (95% CI)	*P*-value
Signature Risk (High vs. Low)	1.63 (1.03-2.61)	0.039
Age (Old vs. Young)	1.8 (1.14-2.83)	0.012
Stage (III/IV vs. I/II)	2.46 (1.55-3.91)	0.0001

N=692; 81 deaths; median f/u: 16.6 months

## DISCUSSION

The objective of the current study was to determine whether miRNAs modulated the metastatic potential of UPS. Through the initial analysis of the miRNA expression pattern from 42 samples constituting the Training Set, we initially identified a collection of miRNAs associated with DMFS (Supplementary Table 1). We observed that these miRNAs were prognostic in other cancer models, including miR-132, miR-143 and miR-181a, which have been previously associated with metastasis in osteosarcoma [20-22]. *In silico* analysis and *in vitro* phenotypic screening assays identified two candidate miRNAs (miR-138 and miR-224) that targeted genes involved in cellular adhesion; hence likely play important roles in mediating UPS metastasis. Experiments of miR-138 over-expression or knock-down confirmed its ability to modulate the previously validated RHOC and ROCK2 target genes [14], thereby supporting a role of miR-138 in the RHO-ROCK-LIMK cell adhesion and motility pathway. Examining published and *in silico* predicted candidates of other DMFS-associated miRNAs suggested that multiple components within this pathway are potentially targeted by these miRNAs, which would advocate for a multifactorial system that modulated metastases. Thus, a multi-miRNA prognostic signature was developed from the Training Set miRNA expression values. The prognostic ability of this 6-miRNA signature was then validated in an independent cohort of UPS, which further corroborated the value of these miRNAs in regulating the metastatic phenotype of this disease.

Three prognostic molecular signatures for STS have been previously published [19, 23, 24]. The “cinsarc” signature comprising of 67 differentially-expressed mRNAs related to cell cycle progression, and chromosomal instability [19] was the sole validated prognostic signature, aside from the miRNA signature in this current report. In addition to the prognostic ability of the cinsarc signature, the group also demonstrated the biological significance of genomic instability in promoting metastases in STS, breast cancer, and lymphoma [19], suggesting that common mechanisms or biological selection may exist between different cancers to promote metastases. None of the 6 miRNAs forming the current miRNA signature are known to directly target the 67 cinsarc genes involved in cell cycle or genomic stability. Thus, beyond genomic instability, other mechanisms might explain, or are the consequence of genomic alterations that lead to the phenotypic aggressiveness of metastatic UPS. Alternatively, no mechanistic or functional association exists between the genes from these two distinct signatures; it is certainly possible that the metastatic ability of STS evolve through multiple means independently.

Our functional study of miRNA-138 and -224 served to validate their targeting of RHOC and ROCK2 within the cell adhesion pathway of UPS cell models. The increased invasion following over-expression of miR-138 (Figure 1), the association between higher miRNA-138 and reduced *RHOA* expression with increased risk of metastasis (Figure 2A, Supplementary Figures 2 and 4D), along with pathway analysis all suggest that the Rho-ROCK pathway is involved in promoting UPS metastasis (Supplementary Figure 4). *In vitro* data confirmed the inhibitory effect of miRNA-138 on RHOC, disinhibiting RHOA to activate downstream effectors ROCK and LIMK (Supplementary Figure 4). These changes also resulted in the observed loss of the spindle shape of pre-miR-138 transfected sarcoma cells (Figure 1A), consistent with previously reported morphological changes secondary to increased RHOA-ROCK activity that enhanced cell migration and invasion in prostate and breast cancer cells [17]. Prior work from sarcoma models [25-28] suggested that migration and invasion necessitated a balance in the activity of different members of the RHO-ROCK pathway, and its complementary rac-associated motility pathway as both amoeboid and mesenchymal cell migration have been associated with increased migration, invasion, and subsequent development of metastasis. The need for subtle and perhaps plastic changes in genes related to cell motility further supports the potential of multifunctional roles for miRNAs in metastases, whereby differential levels of miRNAs would result in marginal alterations of target mRNAs to promote specific phenotypes.

In light of the similar cellular morphology between breast and sarcoma cells following miR-138 and RHOA modulation, we postulated that our six miRNA signature might also be relevant for breast malignancies, and explored its value using the TCGA BRCA dataset [18]. Remarkably, our six-miRNA signature and genes related to the RHO-ROCK adhesion pathway were indeed prognostic for OS in breast cancer, after adjusting for age and stage (Table 3). The association between these genes with breast cancer outcome was further interrogated using the 1056 publically available mRNA profiles from the Combined breast dataset [29-34]. Indeed, low expression of *RHOA* was significantly associated with shorter DMFS (Supplementary Figure 6; *p* = 0.014). These results suggest a common mechanism involving these six miRNAs and *RHOA* in promoting metastasis in both UPS and breast cancer. Contrary to prior reports wherein elevated RHOA and RHOC were considered to be pro-metastatic, involved in epithelial-mesenchymal transition (EMT) [13, 35, 36], our current results derived from sarcoma and breast cancer data suggest that reduced expression of RHOA and RHOC were in fact associated with higher metastatic rates. This unanticipated finding is supported by two recent publications demonstrating the importance of plasticity in cancer cells to undergo EMT followed by a reversion through mesenchymal-epithelial transition (MET) to colonize the target metastatic environment with subsequent proliferation [37, 38]. Increased tumor plasticity may be supported by greater genomic instability observed in more aggressive STS and breast cancers [18, 19]. Furthermore, given the complexity of the metastatic process, miRNA-138 likely partners with other independent molecular aberrations, such as RHOA, other Rho family members, and the other five miRNAs in the UPS signature to further induce and/or promote biological changes in this disease (Supplementary Figure 4C). In concert with the need for tumor plasticity in metastasis, the increased miR-138 and RHOC expression in metastatic samples in comparison with their original primary UPS suggest the decoupling of the miR138-RHOC interaction in the evolution from primary tumors prone to metastasize to the metastatic colonization (Figure 2A). Although the current signature was reasonably successful at dichotomizing patients between “Low” *vs*. “High” risk of developing DM, it is far from achieving 100% accuracy (Figures 2B & 3), likely related to the complex biology, and limitations in the sampling of intra-tumorally heterogeneous tumors.

This study is the first report of a validated miRNA signature that can predict for DMFS (HR = 3.46) in UPS, independent of other known prognostic factors such as age, gender, tumor size, grade, depth, or use of adjuvant treatment (Table 2). The strengths of our study include the focus on a homogeneous subtype of STS, namely UPS, to: a) investigate the prognostic role of miRNA; and b) derive biological understanding of the mechanisms by which STS develop metastases. The role of this signature was further supported by the higher risk scores observed in UPS lung metastases compared to their original primary lesions (Figure 2B), further corroborating the association between this miRNA expression pattern with the development of DM.

While the Union for International Cancer Control staging of STS incorporates tumor depth, size and grade to prognosticate patient groupings, outcomes within each stage remain substantially variable. The current six-miRNA signature was able to predict for DMFS independently of known prognostic factors, thus adds to the prognostication of patients diagnosed with UPS. Identification of a “High-risk” subgroup may advance personalized treatments by selecting these patients for more intensive systemic therapies, subtype specific targeted agents, or research trials. As prolonged survival is achieved through metastasectomies of oligometastatic pulmonary disease, closer monitoring of “High-risk” patients may improve the odds of discovering resectable disease. Before this six-miRNA-signature can be utilized in the clinic however, additional prospective validation trials will be clearly required.

## MATERIALS AND METHODS

### Patients and tissues

Institutional Research Ethics Board approvals were obtained from the University Health Network (UHN) and Mount Sinai Hospital (MSH). Samples were collected from multiple Canadian institutions and stored as fresh frozen tissues within the MSH Clinical Core and Sarcoma Biospecimen Repository, where corresponding clinical data were prospectively annotated. All analyzed samples underwent a central pathology review by a sarcoma pathologist (BD), and contained more than 70% tumor cells. The Training Set comprised of 42 pre-treatment primary UPS samples from Stage I-III patients diagnosed from 1988-1999 (Table 1). The Validation Set comprised of a similar cohort of primary samples from 68 Stage I-III UPS patients diagnosed from 2000-2010. Resected lung metastases (*n* = 10 samples) derived from six patients in the Validation Set were also obtained. Our cohort of patients were divided into Training and Validation sets based on the date of tissue collection whereby the Training Set were derived from patients treated during the first 11 years of tissue banking, and the Validation Set originated from samples collected in the more recent 11 years. Throughout these time periods, the overall management of sarcomas has not changed, nor has the clinical outcome of these patients.

RNA from normal mesenchymal tissues (smooth muscle (Clontech Laboratories, Mountain View, CA); adipose tissue (Applied Biosystem, Carlsbad, CA); carotid artery and, vein (Agilent Technologies, Santa Clara, CA)) were co-profiled for their miRNA expressions at the same time as the Training Set samples

### miRNA analyses

All samples were assayed randomly and blinded to study endpoints to avoid experimental bias. Global profiling of miRNA expression on the Training Set was performed using TaqMan^®^ Human Micro-RNA Array-A (Applied Biosystems). Total RNA (300 ng) was first reverse-transcribed with the Multiplex RT pool set, then quantitated using an Applied Biosystems 7900 HT Real-Time PCR system, as previously described [39]. Data were normalized using endogenous controls (RNU6B, RNU44, and RNU48) that were simultaneously quantified. The resulting ΔC_T_ expression values were used for hierarchical clustering (JMP10-SAS Institute, Cary, NC), and signature derivation. Quantification of the signature miRNAs in the Validation Set was performed using single well qRT-PCR by initially reverse-transcribing 200 ng of total RNA with multiscribe reverse transcriptase and miRNA-specific primers (50 nM), followed by qRT-PCR analysis using TaqMan microRNA Assays (Applied Biosystems) [39]. Determination of miRNA ΔC_T_:

*n:* sample number

*C_T_(X_n_), C_T_(Y_n_), C_T_(Z_n_):* raw C_T_ value in well X, Y and Z for sample *n* performed in triplicates for each miRNA and endogenous control investigated.

miR-132ΔC_Tn_ = [average (miR-132 *C_T_(X_n_), (Y_n_), (Z_n_)*)] − [average ( average (RNU6B *C_T_(X_n_), (Y_n_), (Z_n_)*), average (RNU48 *C_T_(X_n_), (Y_n_), (Z_n_)*))]

The calculations were repeated using the same formula for the other miRNAs (miR-138, miR-143, miR-221, miR-224, and miR-491-5p).

### Survival analyses

All clinical endpoints were calculated from the date of surgery to the event date or last follow-up date if no event had occurred. For the primary endpoint, DMFS events comprised of DM or death. Overall survival (OS) events comprised of death from any cause. Disease-free survival (DFS) events were defined by any local or distant relapse, or death. All samples were successfully profiled for miRNA expression; one sample from the Training Set was missing the event date of DM.

Univariate analyses were conducted on the Training Set using Cox Proportional Hazards (PH) regression model to determine candidate miRNAs (*p*-value < 0.1) for multivariate analysis. Cox PH multivariate regression model was fitted onto the Training Set using stepwise selection with the significance level set at 0.1, and DMFS as the primary outcome in order to select a small set of prognostic miRNAs. The signature score was based on the weighted combination of the expressions (ΔC_T_) of the miRNAs selected at the previous step, with the estimated regression coefficients of the Cox PH regression model as the weights [40, 41]. Hazards Ratios (HRs) and 95% Confidence Intervals (CIs) were estimated for significant predictors of DMFS, DFS and OS. Multivariate analysis for the signature included known prognostic factors (patient age, tumor maximum size, grade, depth), gender, and administration of radiotherapy. Statistical significance level for the Validation Set analysis was < 0.05. Statistics were performed using SAS version 9.3 (SAS Institute), and the R statistical programming environment (R Project Foundation).

### RNA analyses

Quantitative RT-PCR was utilized to analyze mRNA expression of *RHOA*, *RHOC*, *ROCK1*, *ROCK2*, *LIMK1*, and *GAPDH*. Following reverse transcription of 200 ng of total RNA using SuperScript III Reverse Transcriptase (Invitrogen), qRT-PCR was performed using SYBR Green PCR Master Mix (Applied Biosystems). Relative mRNA levels were calculated using the 2−ΔΔ*Ct* method [42]. Primers for PCR amplifications were designed using Primer3 Input 0.4.0 and as followed: RHOA (Forward 5′-AAGGACCAGTTCCCAGAGGT-3′; Reverse 5′-TTCTGGGGTCCACTTTTCTG-3′), RHOC (Forward 5′-GAGAGCTGGCCAAGATGAAG-3′; Reverse 5′-TTGGGGATCTCAGAGAATGG-3′), ROCK1 (Forward 5′-ACGGGACAAAATGGGAGAGT-3′; Reverse 5′-ACAAGGGAGGGAGAAGAGGA-3′), ROCK2 (Forward 5′-AGAACCTGTCAAGCGTGGTA-3′; Reverse 5′-CAAGGCTTGGAGTTGTGACC-3′), LIMK1 (Forward 5′-TGTAGCCACAGAGGATGCTG-3′; Reverse 5′-TGAGGCAGATGAAACACTCG-3′), and GAPDH (Forward 5′-AGTCAACGGATTTGGTCGT-3′; Reverse 5′-TTGATTTTGGAGGGATCTCG-3′).

### Cell lines and reagents

Four primary sarcoma cell lines (STS48, STS93, STS109, and STS117) derived from patients diagnosed with UPS were utilized for the *in vitro* experiments. Cell lines were tested and authenticated using Short Tandem Repeat (STR) profiling of the following loci for each cell line: Amelogenin, CSF1PO, D13S317, D16S539, D18S51, D19S433, D21S11, D2S1338, D3S1358, D5S818, D7S820, D8S1179, FGA, THO1, TPOX, and vWA. Cells were tested on the second passage after the initial receipt from the laboratory of RG. All *in vitro* experiments were performed using cells within 20 passages from the time of STR testing.

Cells were incubated at 37°C under 5% CO_2_ in DMEM:F12 1:1 media with 10% bovine serum. Lipofectamine-2000 (Invitrogen, Carlsbad, CA) was used to transfect cells with pre-miRs (Invitrogen), Locked-Nucleic-Acid (LNA) anti-miRs (Exiqon), or siRNAs (Qiagen). Total RNA from samples were extracted using RNeasy kits (Qiagen). Recovered RNA concentration and quality were measured using the Nanodrop 1000A spectrophotometer (Nanodrop Technologies, Wilmington, DE).

### Clonogenic and cell cycle assays

Cellular effects of transfections were measured using clonogenic assays [43]; cell cycle analyses of transfected cells were performed using propidium iodide staining [43]. Analyses were conducted with the BD FACScalibur using the FL-2 channel; flow cytometry data were analyzed using FlowJo software (Tree Star).

### Migration and invasion assays

Cells were initially transfected with 10 nM pre-miR-138, 10 nM pre-miR-scrambled control, 50 nM Locked Nucleic Acid (LNA) miR-138, or 50 nM LNA-scrambled control. After 48 hours, cells were trypsinized, then 1.5×10^5^ cells from each condition were seeded inside the Control or Matrigel coated trans-well chambers (BD Bioscience, Franklin Lakes, NJ) with RPMI 1640 medium containing 1% bovine serum. The chambers were then placed in 24-well plates containing RPMI 1640 medium, and 20% bovine serum served as the chemo-attractant for the cells. After incubating for 24 hours, cells were fixed and stained using the Diff Quik® Set (Siemens Healthcare Diagnostics Inc., Deerfield, IL); then the number of cells that invaded to the opposite side of the membrane was counted under a microscope. Results were analyzed to derive each condition's invasion index, which represented the invasive ability of the cells over their migration ability, calculated as (Invasion/Migration of Test Cell)/(Invasion/Migration of Control Cell) for each condition. This analytical method thereby accounted for any cytotoxic effects from pre-miR or LNA transfections.

### Gene expression array after miRNA modulation

Global gene expression was profiled using Affymetrix Human Genome U133 plus 2.0 Array (Affymetrix, Inc., Santa Clara, CA), performed at the Ontario Cancer Institute Genomics Centre using 100 ng of total RNA to determine the change in gene expression following transfection with control LNA, LNA miR-138, or LNA miR-224. The arrays were hybridized for 17 hrs at 45°C, then washed and stained in fluidic station P450. Images were acquired with GeneChip scanner 3000, and preliminary analysis was conducted with the Affymetrix gene expression console. Data were pre-processed using the RMA method, and 3X median for background removal.

### Western blot

Monoclonal rabbit antibodies for western analysis were all purchased from Cell Signaling Technology Inc., Danvers, MA. These included the antibodies for RhoA, RhoC, ROCK1, ROCK2, LIMK1, LIMK2, and Phospho-LIMK1 (Thr508)/LIMK2 (Thr505). Protein extraction, immunoblotting, secondary antibody conjugation, and western blot quantification techniques were performed as previously described [44].

### Statistical analysis of *in vitro* experiments

All *in vitro* experiments were conducted in triplicate at least three independent times. Statistical differences between treatment groups were determined using a Student *t-*test.

The Cancer Genome Atlas (TCGA) breast cancer BRCA dataset was utilized, comprising of 762 samples with corresponding miRNA and mRNA profilings [18]. MiRNA counts-per-million-reads and mRNA reads-per-kilobase-per-million-reads from individual datasets were converted into z-scores. The six-miRNA-signature score was then applied onto the z-scores to dichotomize patients into risk groups (High *vs*. Low). Six other breast cancer datasets (*n* = 1056) [29-34] were utilized for assessment of RhoA mRNA expression in relation to DMFS. These datasets were selected for their use of Affymetrix mRNA profiling platforms with annotated DMFS outcome. Transcript mRNA expressions were converted into z-scores within each dataset, then combined together for analysis; henceforth denoted as Combined breast dataset (BRCA). The association of the 6-miRNA-signature and mRNA expression with clinical outcome was examined using multivariate Cox PH models while adjusting for age and disease stage. Univariate analysis of the correlation between BRCA patient OS, and tumor mRNA expression of the RHO-ROCK-LIMK adhesion pathway was also performed. Interaction analysis between BRCA 6-miRNA-signature score with the expression of mRNAs from the RHO-ROCK-LIMK pathway, and estrogen receptor status was explored using Chi-Square tests.

## SUPPLEMENTARY MATERIAL FIGURES AND TABLES


