# Supplementary material for: Prognostic microRNAs modulate the RHO adhesion pathway: A potential therapeutic target in undifferentiated pleomorphic sarcomas
Source: Oncotarget. 2015 Apr 23;6(36):39127–39. doi: 10.18632/oncotarget.3926 (PMC4770761; doi:10.18632/oncotarget.3926)
Supplement: Supplementary file 1 [file oncotarget-06-39127-s001.pdf]

# Prognostic microRNAs modulate the RHO adhesion pathway: A potential therapeutic target in undifferentiated pleomorphic sarcomas

## Supplementary Material

### SUPPLEMENTARY FIGURE 1

Unsupervised hierarchical clustering using the Ward method of miRNA expression from 4 Normal tissues (Adipose, Carotid, Vein and Smooth Muscle) (Purple), 4 primary UPS cell lines (STS48, STS93, STS109 and STS117) (Blue) and 42 primary UPS samples from the Training Set that metastasized (Yellow) or that did not-metastasize (White).

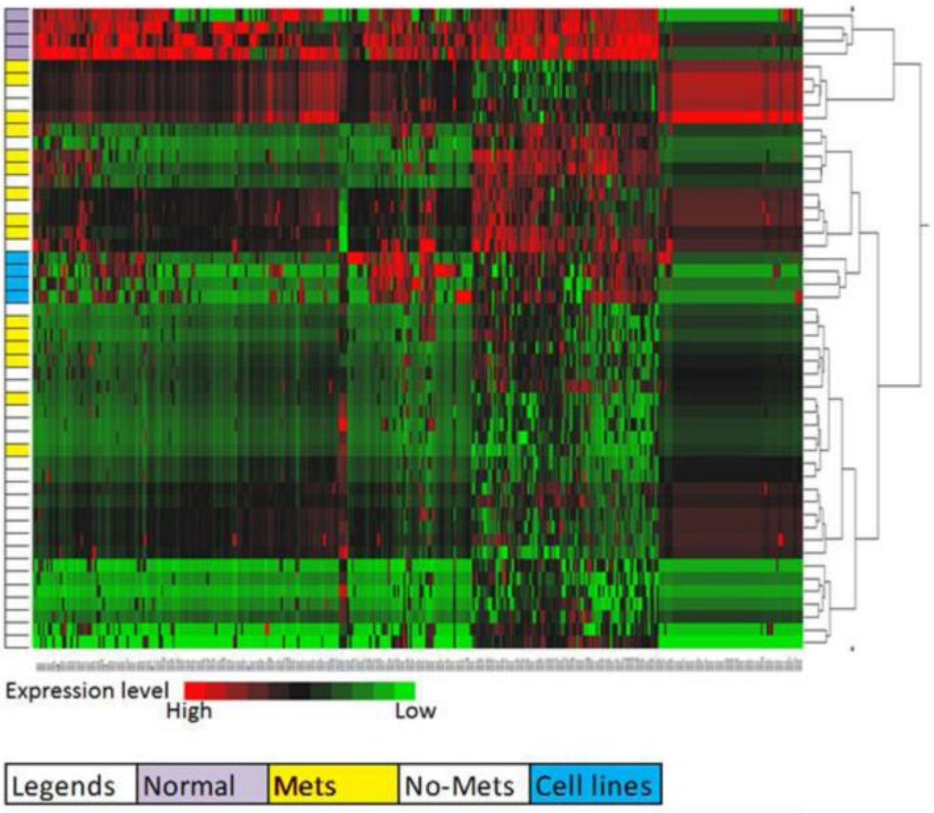

## SUPPLEMENTARY FIGURE 2

Univariate association of miR-138 and miR-224 expression with A) distant metastasis free survival (DMFS) and B) disease free survival (DFS) in the combined UPS cohorts (Training Set + Validation Set). C) Multivariate analysis of the associations of individual miRNAs from the 6-miRNA signature with DMFS and DFS in the combined UPS cohorts of Training Set and Validation Set. Combined datasets were used to obtain sufficient events for multivariate analyses. Values of miRNA expressions were dichotomized by the median ΔCt values. Multivariate adjustment was performed using 6 clinical factors (patient age, tumor size, grade, depth, gender and radiotherapy use).

**A)**

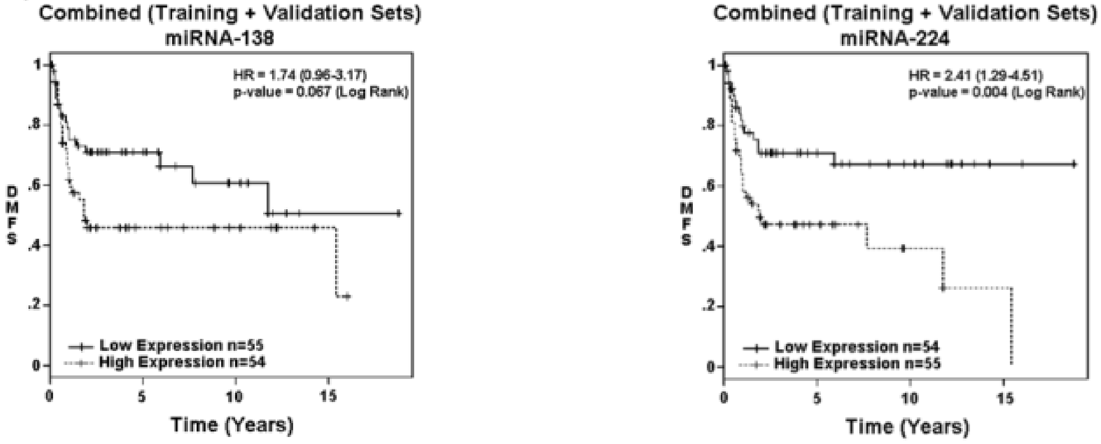


**B)**

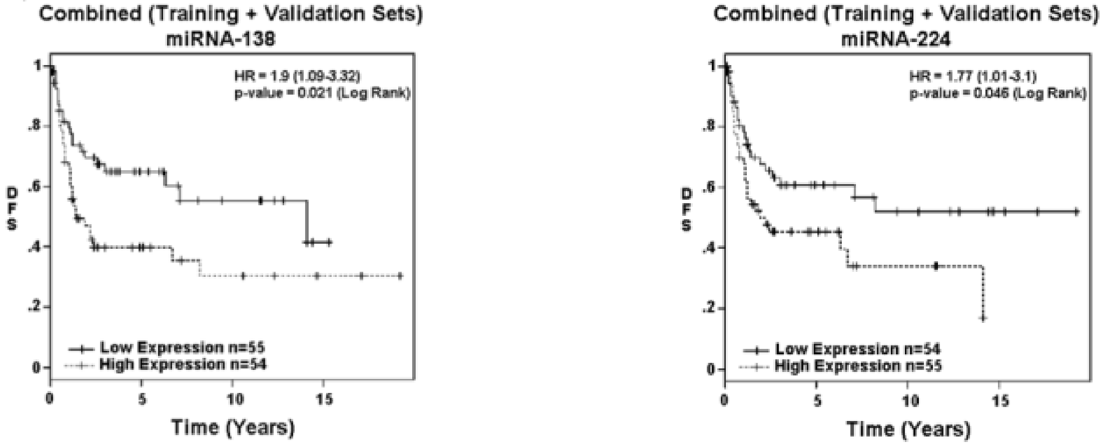


**C)**

| | | Multivariate Analysis for DMFS | | Multivariate Analysis for DFS | |
|---|---|---|---|---|---|
| | | **Hazard Ratio (95% CI)** | **P-value** | **Hazard Ratio (95% CI)** | **P-value** |
| Mir-132 | Low vs. High | 0.99 (0.51-1.9) | 0.97 | 1.06 (0.58-1.94) | 0.85 |
| Mir-138 | Low vs. High | 2.62 (1.38-4.99) | **0.003** | 2.47 (1.36-4.47) | **0.003** |
| Mir-143 | Low vs. High | 1.7 (0.91-3.2) | 0.097 | 1.43 (0.81-2.54) | 0.22 |
| Mir-221 | Low vs. High | 1.17 (0.64-2.14) | 0.62 | 1.08 (0.62-1.88) | 0.78 |
| Mir-224 | Low vs. High | 2.18 (1.12-4.21) | **0.021** | 1.76 (0.98-3.18) | 0.06 |
| Mir-491-5p | Low vs. High | 0.86 (0.45-1.63) | 0.63 | 0.83 (0.46-1.49) | 0.53 |

**SUPPLEMENTARY FIGURE 3**

Clonogenic assay of STS117 cells following transfection with 5 nM and 50 nM of Locked Nucleic Acid (miR-138 and miR-224). Data are presented as the mean $\pm$ standard error of the mean.

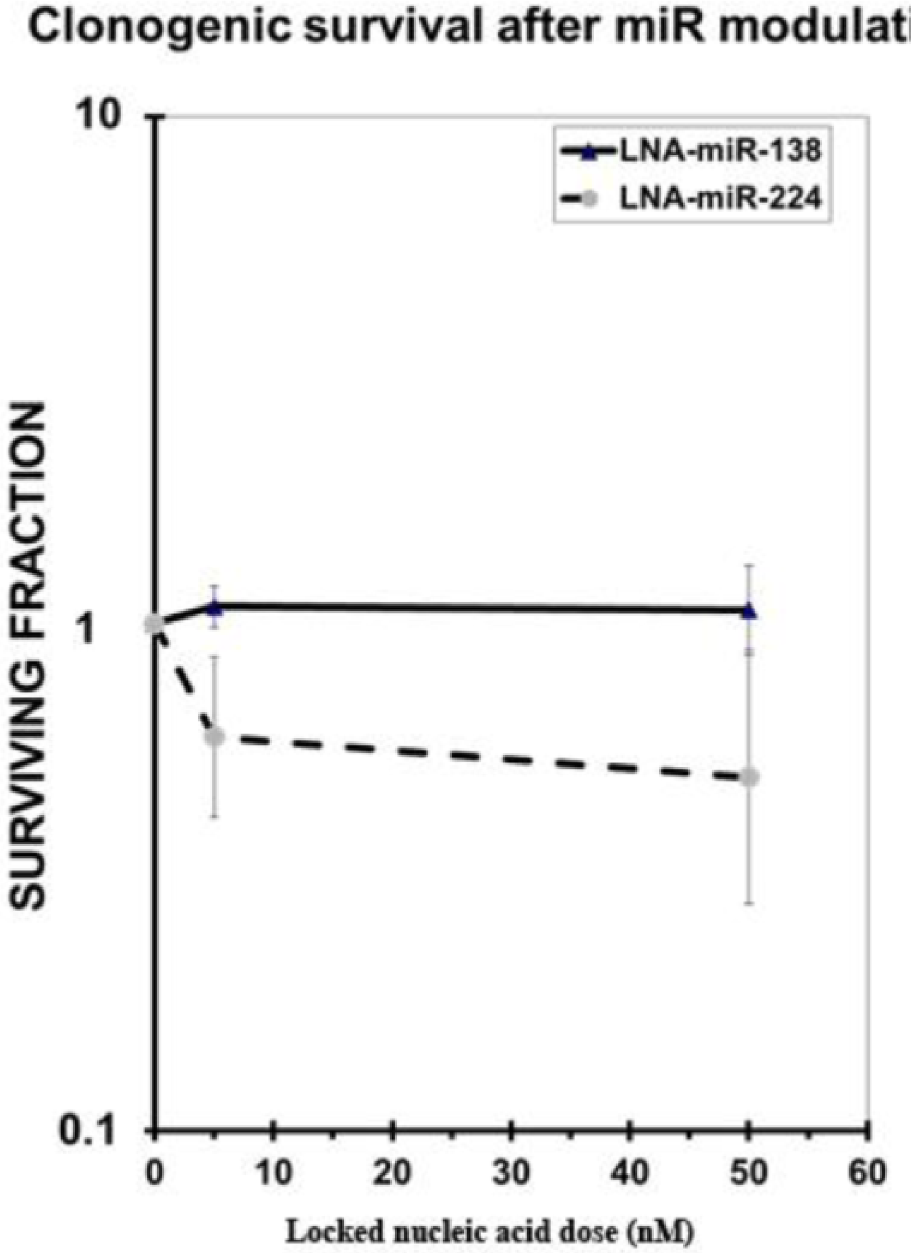

## SUPPLEMENTARY FIGURE 4

Quantification in STS117 and STS48 of A) RhoC and ROCK2 mRNA levels following miR-138 modulation using pre-miR-138 or Locked nucleic acid (LNA) miR-138 demonstrating significant reduction in RhoC and ROCK2 expression with pre-miR-138 in relation to cells transfected with control (scrambled) pre-miR or LNA. B) Corresponding protein level and activity downstream of miR-138 and RhoA were assessed following transfection with pre-miR-control, pre-miR-138, LNA-miR-control and LNA-miR-138. C) A simplified Rho-ROCK-LIMK adhesion pathway depicts the targets of miR-138 (RhoC and ROCK2) and their interactions with RhoA and ROCK1. After binding with RhoA or RhoC, ROCK1 and ROCK2 can then activate the kinase activity of LIMK1 and LIMK2 through phosphorylation. The activity of Cofilin (1 and 2), a protein essential to actin fiber remodeling, is mediated by activated LIMKs and slingshot phosphatases (SSH1, SSH2 and SSH3). D) RhoA mRNA expression in primary UPS samples from patients who had no metastasis (Primary No DM) (n=14) vs. primary UPS which developed metastases (Primary DM) (n=14) vs. lung metastatic samples (Metastasis) (n=10). Significant differences in RhoA mRNA expression were observed between each group (Student t-test $p \leq 0.006$). Data plotted as ΔΔCt normalized to the average values from the No-DM group (Lower values = Lower expression). MRNA expression data presented as the mean ± standard error of the mean.

A)

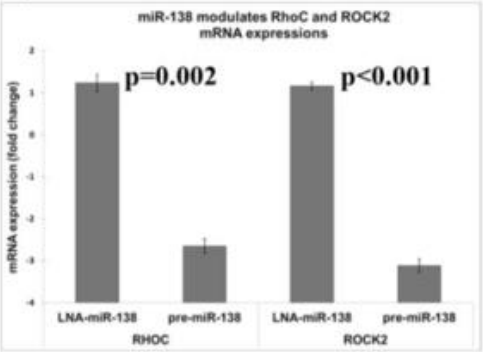


B)

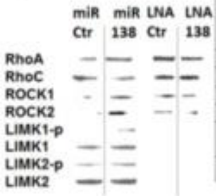


| | pre-miR Control | pre-miR 138 | LNA-miR Control | LNA-miR 138 |
|---|---|---|---|---|
| RhoA | 1 | 1.34 | 1 | 0.68 |
| RhoC | 1 | 0.48 | 1 | 1.24 |
| ROCK1 | 1 | 4.29 | 1 | 0.38 |
| ROCK2 | 1 | 26.9 | 1 | 0.34 |
| P-LIMK1:LIMK1 | 1 | 12 | | |
| P-LIMK2:LIMK2 | 1 | 2.5 | | |

C)

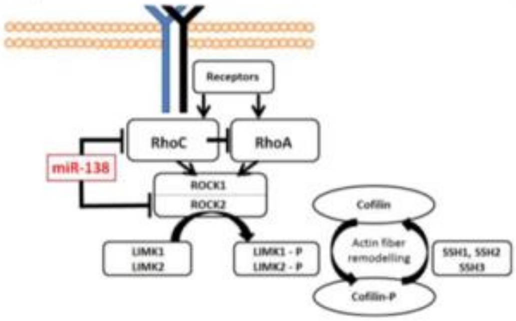


D)

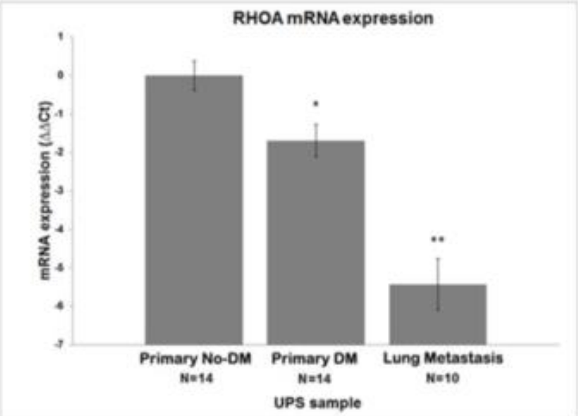


* p=0.006 (Primary No DM vs. Primary DM)

** p<0.001 (Primary No DM vs. Metastasis; Primary DM vs. Metastasis)

**SUPPLEMENTARY FIGURE 5**

Overall survival of patients from the Training Set and Validation Set.

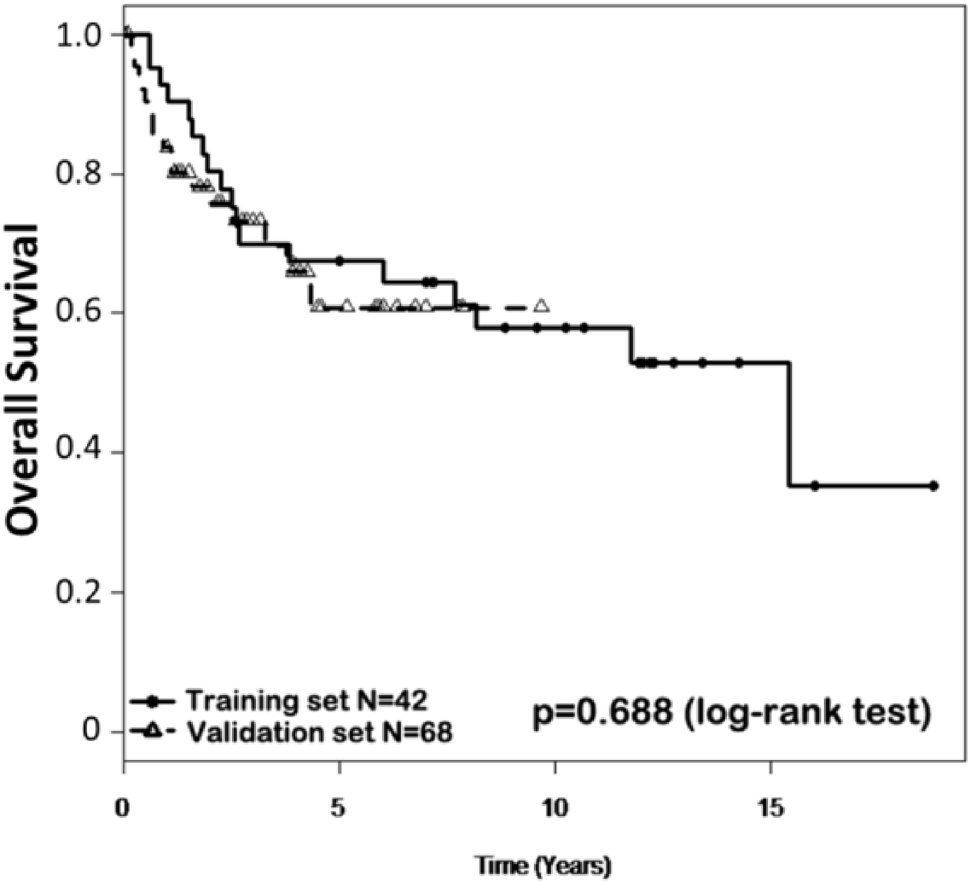

**SUPPLEMENTARY FIGURE 6**

Distant metastasis free survival (DMFS) of 1056 breast cancer patients from the Combined breast dataset dichotomized by the median expression of RhoA. Median follow-up was 238 months.

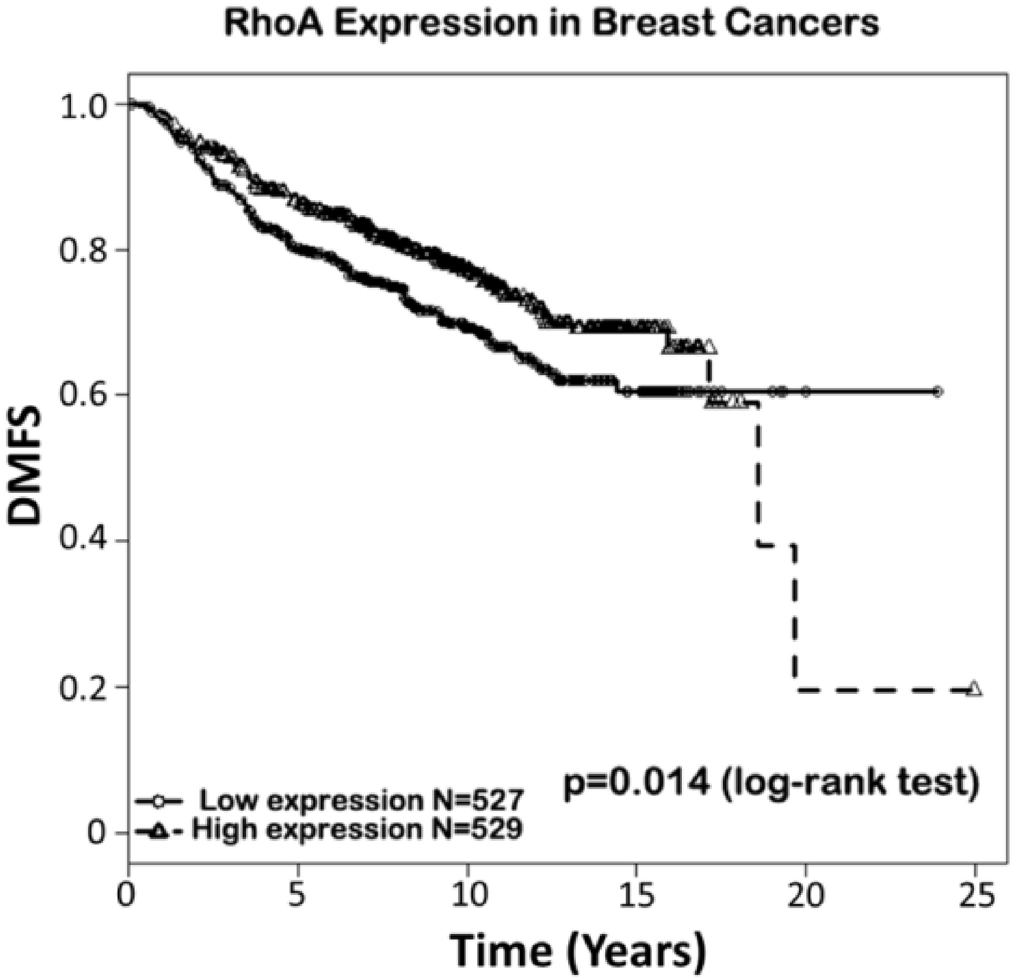

**SUPPLEMENTARY TABLE 1: MiRNAs were associated with distant metastasis free survival**

Forty miRNAs were associated with distant metastasis free survival (DMFS) for the patients in the Training Set. These miRNAs are listed below according to the significance of the association (ascending p-value) with DMFS. Each miRNA's concordance index (C-index) with DMFS, difference in expression between samples that developed metastasis (DM) and those that did not metastasize (noDM), and its prognostic associations in cancers are listed. Thirty-one of the miRNAs potentially target 113 genes involved in the focal adhesion pathway (DIANA miRpath); the number of focal adhesion pathway genes potentially targeted by each miRNA is indicated in the last column and in the figure.

| miRNA | P-value | C-index | $\Delta\Delta C_T$ (DM – noDM) | Prognostic association with cancers (refs) | Number of genes in focal adhesion pathway |
|---|---|---|---|---|---|
| miR-491-5p | 0.003 | 0.702 | 1.786 | Oral(1) | 2 |
| miR-224 | 0.004 | 0.686 | 2.298 | Breast, Cervix, Colorectal, Germ cell tumor, Medulloblastoma Prostate, Pancreas(2-9) | 8 |
| miR-130a | 0.005 | 0.677 | 0.400 | Endometrial, Gastric, Lung(10-12) | 16 |
| miR-128 | 0.005 | 0.626 | 0.773 | | 23 |
| miR-886-5p | 0.011 | 0.629 | 0.829 | | |
| miR-204 | 0.012 | 0.557 | 1.670 | Melanoma(13) | 6 |
| miR-324-3p | 0.013 | 0.648 | 0.582 | Melanoma(14) | |
| miR-197 | 0.02 | 0.609 | 1.157 | Breast, Lung(15, 16) | 1 |
| miR-99b | 0.021 | 0.633 | 0.673 | Esophageal(17) | |
| miR-744 | 0.024 | 0.665 | 1.036 | | 1 |
| miR-339-3p | 0.027 | 0.642 | 0.389 | | |
| miR-512-3p | 0.027 | 0.608 | 0.856 | | 7 |
| miR-15a | 0.027 | 0.608 | 0.910 | Larynx(18) | 21 |
| miR-23b | 0.028 | 0.608 | 0.899 | | 1 |
| miR-202 | 0.028 | 0.619 | 1.297 | | 7 |
| miR-455-3p | 0.029 | 0.654 | 0.892 | | |
| miR-200c | 0.032 | 0.613 | 0.746 | Colorectal, Esophageal, Hepatocellular, Melanoma(19-22) | 1 |
| miR-10a | 0.034 | 0.641 | 0.388 | Renal(23) | 3 |
| miR-139-5p | 0.037 | 0.617 | 0.326 | | 2 |
| miR-132 | 0.037 | 0.613 | 0.492 | Osteosarcoma(24) | 1 |
| miR-212 | 0.041 | 0.573 | -0.04 | | 10 |
| miR-374a | 0.041 | 0.641 | 0.508 | Breast(25) | 18 |
| miR-486-3p | 0.042 | 0.578 | 2.746 | | 6 |
| miR-148b | 0.047 | 0.611 | 0.502 | Breast, Pancreas(26, 27) | 8 |

| | | | | | |
|---|---|---|---|---|---|
| let-7c | 0.047 | 0.633 | 1.057 | Cervix, Lung(28, 29) | 14 |
| miR-181a | 0.049 | 0.585 | 0.572 | Osteosarcoma(30) | 7 |
| miR-221 | 0.051 | 0.656 | 1.234 | Breast, Colorectal, Gastric, Pancreas, Prostate, Renal, Thyroid(8, 10, 31-39) | 5 |
| miR-143 | 0.057 | 0.651 | 0.535 | Cervix, Colorectal, Esophageal, Osteosarcoma, Pancreas, Prostate(28, 38, 40-44) | 3 |
| miR-106b | 0.058 | 0.673 | 0.827 | Hepatocellular, Renal(38, 42) | 13 |
| miR-125b | 0.059 | 0.613 | 0.798 | Breast, Cervix, Gastric, Hepatocellular, Lung, Melanoma(25, 28, 45-51) | 6 |
| miR-138 | 0.062 | 0.573 | 1.679 | | 5 |
| miR-886-3p | 0.062 | 0.578 | 1.330 | Lung(52) | |
| miR-142-3p | 0.062 | 0.599 | 0.443 | Esophageal(53) | 4 |
| miR-331-3p | 0.063 | 0.567 | 0.442 | | |
| miR-375 | 0.078 | 0.56 | 1.609 | Breast, Esophageal, Head and neck, Lung, Nasopharynx, Prostate, Thyroid(54-63) | 3 |
| miR-517a | 0.083 | 0.585 | 0.236 | | |
| miR-598 | 0.083 | 0.566 | 1.056 | | |
| miR-93 | 0.085 | 0.654 | 1.546 | Cervix, Colorectal, Gastric, Lung(10, 64-66) | 5 |
| miR-198 | 0.089 | 0.576 | 1.694 | | 2 |
| miR-452 | 0.098 | 0.539 | 1.413 | Urothelial(67) | 6 |

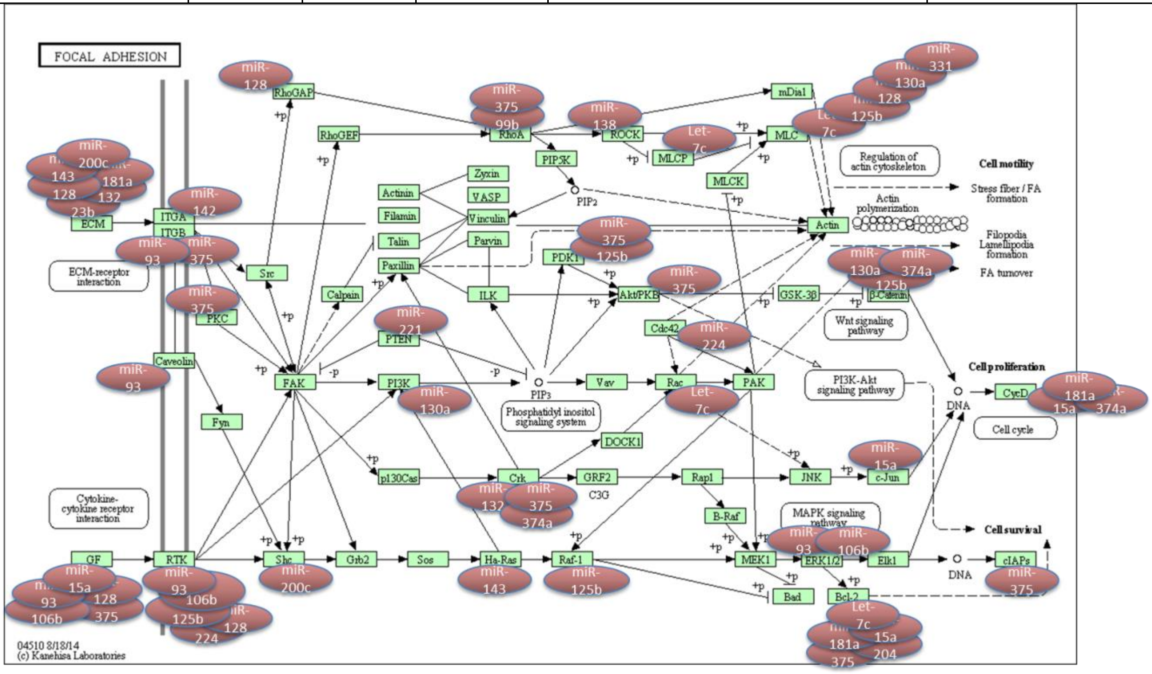

1. Huang WC, Chan SH, Jang TH, Chang JW, Ko YC, Yen TC, et al. miRNA-491-5p and GIT1 serve as modulators and biomarkers for oral squamous cell carcinoma invasion and metastasis. Cancer Res. 2014;74:751-64.
2. Gokhale A, Kunder R, Goel A, Sarin R, Moiyadi A, Shenoy A, et al. Distinctive microRNA signature of medulloblastomas associated with the WNT signaling pathway. J Cancer Res Ther. 2010;6:521-9.
3. Huang L, Dai T, Lin X, Zhao X, Chen X, Wang C, et al. MicroRNA-224 targets RKIP to control cell invasion and expression of metastasis genes in human breast cancer cells. Biochem Biophys Res Commun. 2012;425:127-33.
4. Lin ZY, Huang YQ, Zhang YQ, Han ZD, He HC, Ling XH, et al. MicroRNA-224 inhibits progression of human prostate cancer by downregulating TRIB1. Int J Cancer. 2014;135:541-50.
5. Mees ST, Mardin WA, Sielker S, Willscher E, Senninger N, Schleicher C, et al. Involvement of CD40 targeting miR-224 and miR-486 on the progression of pancreatic ductal adenocarcinomas. Ann Surg Oncol. 2009;16:2339-50.
6. Mosakhani N, Pazzaglia L, Benassi MS, Borze I, Quattrini I, Picci P, et al. MicroRNA expression profiles in metastatic and non-metastatic giant cell tumor of bone. Histol Histopathol. 2013;28:671-8.
7. Shen SN, Wang LF, Jia YF, Hao YQ, Zhang L, Wang H. Upregulation of microRNA-224 is associated with aggressive progression and poor prognosis in human cervical cancer. Diagn Pathol. 2013;8:69.
8. Tang J, Ahmad A, Sarkar FH. The Role of MicroRNAs in Breast Cancer Migration, Invasion and Metastasis. Int J Mol Sci. 2012;13:13414-37.
9. Yuan K, Xie K, Fox J, Zeng H, Gao H, Huang C, et al. Decreased levels of miR-224 and the passenger strand of miR-221 increase MBD2, suppressing maspin and promoting colorectal tumor growth and metastasis in mice. Gastroenterology. 2013;145:853-64 e9.
10. Kim BH, Hong SW, Kim A, Choi SH, Yoon SO. Prognostic implications for high expression of oncogenic microRNAs in advanced gastric carcinoma. J Surg Oncol. 2013;107:505-10.
11. Li BL, Lu W, Lu C, Qu JJ, Yang TT, Yan Q, et al. CpG island hypermethylation-associated silencing of microRNAs promotes human endometrial cancer. Cancer Cell Int. 2013;13:44.
12. Wang XC, Tian LL, Wu HL, Jiang XY, Du LQ, Zhang H, et al. Expression of miRNA-130a in nonsmall cell lung cancer. Am J Med Sci. 2010;340:385-8.
13. Jukic DM, Rao UN, Kelly L, Skaf JS, Drogowski LM, Kirkwood JM, et al. Microrna profiling analysis of differences between the melanoma of young adults and older adults. J Transl Med. 2010;8:27.
14. Greenberg E, Besser MJ, Ben-Ami E, Shapira-Frommer R, Itzhaki O, Zikich D, et al. A comparative analysis of total serum miRNA profiles identifies novel signature that is highly indicative of metastatic melanoma: a pilot study. Biomarkers. 2013;18:502-8.
15. Volinia S, Galasso M, Sana ME, Wise TF, Palatini J, Huebner K, et al. Breast cancer signatures for invasiveness and prognosis defined by deep sequencing of microRNA. Proc Natl Acad Sci U S A. 2012;109:3024-9.
16. Zheng D, Haddadin S, Wang Y, Gu LQ, Perry MC, Freter CE, et al. Plasma microRNAs as novel biomarkers for early detection of lung cancer. Int J Clin Exp Pathol. 2011;4:575-86.
17. Feber A, Xi L, Pennathur A, Gooding WE, Bandla S, Wu M, et al. MicroRNA prognostic signature for nodal metastases and survival in esophageal adenocarcinoma. Ann Thorac Surg. 2011;91:1523-30.
18. Li L, Zhang ZM, Liu Y, Wei MH, Xue LY, Zou SM, et al. [DNA microarrays-based microRNA expression profiles derived from formalin-fixed paraffin-embedded tissue blocks of squammous cell carcinoma of larynx]. Zhonghua Bing Li Xue Za Zhi. 2010;39:391-5.
19. Liu S, Tetzlaff MT, Cui R, Xu X. miR-200c inhibits melanoma progression and drug resistance through down-regulation of BMI-1. Am J Pathol. 2012;181:1823-35.
20. Toiyama Y, Hur K, Tanaka K, Inoue Y, Kusunoki M, Boland CR, et al. Serum miR-200c is a novel prognostic and metastasis-predictive biomarker in patients with colorectal cancer. Ann Surg. 2014;259:735-43.
21. Yeh TS, Wang F, Chen TC, Yeh CN, Yu MC, Jan YY, et al. Expression profile of microRNA-200 family in hepatocellular carcinoma with bile duct tumor thrombus. Ann Surg. 2014;259:346-54.

22. Zhao BS, Liu SG, Wang TY, Ji YH, Qi B, Tao YP, et al. Screening of microRNA in patients with esophageal cancer at same tumor node metastasis stage with different prognoses. Asian Pac J Cancer Prev. 2013;14:139-43.
23. Wu X, Weng L, Li X, Guo C, Pal SK, Jin JM, et al. Identification of a 4-microRNA signature for clear cell renal cell carcinoma metastasis and prognosis. PLoS One. 2012;7:e35661.
24. Yang J, Gao T, Tang J, Cai H, Lin L, Fu S. Loss of microRNA-132 predicts poor prognosis in patients with primary osteosarcoma. Mol Cell Biochem. 2013;381:9-15.
25. Cascione L, Gasparini P, Lovat F, Carasi S, Pulvirenti A, Ferro A, et al. Integrated microRNA and mRNA signatures associated with survival in triple negative breast cancer. PLoS One. 2013;8:e55910.
26. Cimino D, De Pitta C, Orso F, Zampini M, Casara S, Penna E, et al. miR148b is a major coordinator of breast cancer progression in a relapse-associated microRNA signature by targeting ITGA5, ROCK1, PIK3CA, NRAS, and CSF1. FASEB J. 2013;27:1223-35.
27. Zhao G, Zhang JG, Liu Y, Qin Q, Wang B, Tian K, et al. miR-148b functions as a tumor suppressor in pancreatic cancer by targeting AMPKalpha1. Mol Cancer Ther. 2013;12:83-93.
28. Huang L, Lin JX, Yu YH, Zhang MY, Wang HY, Zheng M. Downregulation of six microRNAs is associated with advanced stage, lymph node metastasis and poor prognosis in small cell carcinoma of the cervix. PLoS One. 2012;7:e33762.
29. Zhao B, Han H, Chen J, Zhang Z, Li S, Fang F, et al. MicroRNA let-7c inhibits migration and invasion of human non-small cell lung cancer by targeting ITGB3 and MAP4K3. Cancer Lett. 2014;342:43-51.
30. Jones KB, Salah Z, Del Mare S, Galasso M, Gaudio E, Nuovo GJ, et al. miRNA signatures associate with pathogenesis and progression of osteosarcoma. Cancer Res. 2012;72:1865-77.
31. Chou CK, Chen RF, Chou FF, Chang HW, Chen YJ, Lee YF, et al. miR-146b is highly expressed in adult papillary thyroid carcinomas with high risk features including extrathyroidal invasion and the BRAF(V600E) mutation. Thyroid. 2010;20:489-94.
32. Falkenberg N, Anastasov N, Rappl K, Braselmann H, Auer G, Walch A, et al. MiR-221/-222 differentiate prognostic groups in advanced breast cancers and influence cell invasion. Br J Cancer. 2013;109:2714-23.
33. Heinzelmann J, Henning B, Sanjmyatav J, Posorski N, Steiner T, Wunderlich H, et al. Specific miRNA signatures are associated with metastasis and poor prognosis in clear cell renal cell carcinoma. World J Urol. 2011;29:367-73.
34. Jikuzono T, Kawamoto M, Yoshitake H, Kikuchi K, Akasu H, Ishikawa H, et al. The miR-221/222 cluster, miR-10b and miR-92a are highly upregulated in metastatic minimally invasive follicular thyroid carcinoma. Int J Oncol. 2013;42:1858-68.
35. Kawaguchi T, Komatsu S, Ichikawa D, Morimura R, Tsujiura M, Konishi H, et al. Clinical impact of circulating miR-221 in plasma of patients with pancreatic cancer. Br J Cancer. 2013;108:361-9.
36. Liu K, Li G, Fan C, Diao Y, Wu B, Li J. Increased Expression of MicroRNA-221 in gastric cancer and its clinical significance. J Int Med Res. 2012;40:467-74.
37. Spahn M, Kneitz S, Scholz CJ, Stenger N, Rudiger T, Strobel P, et al. Expression of microRNA-221 is progressively reduced in aggressive prostate cancer and metastasis and predicts clinical recurrence. Int J Cancer. 2010;127:394-403.
38. Tokarz P, Blasiak J. The role of microRNA in metastatic colorectal cancer and its significance in cancer prognosis and treatment. Acta Biochim Pol. 2012;59:467-74.
39. Zhou YL, Liu C, Dai XX, Zhang XH, Wang OC. Overexpression of miR-221 is associated with aggressive clinicopathologic characteristics and the BRAF mutation in papillary thyroid carcinomas. Med Oncol. 2012;29:3360-6.
40. Mayne GC, Hussey DJ, Watson DI. MicroRNAs and esophageal cancer--implications for pathogenesis and therapy. Curr Pharm Des. 2013;19:1211-26.
41. Mekenkamp LJ, Tol J, Dijkstra JR, de Krijger I, Vink-Borger ME, van Vliet S, et al. Beyond KRAS mutation status: influence of KRAS copy number status and microRNAs on clinical outcome to cetuximab in metastatic colorectal cancer patients. BMC Cancer. 2012;12:292.

42. Ouyang L, Liu P, Yang S, Ye S, Xu W, Liu X. A three-plasma miRNA signature serves as novel biomarkers for osteosarcoma. Med Oncol. 2013;30:340.
43. Peng X, Guo W, Liu T, Wang X, Tu X, Xiong D, et al. Identification of miRs-143 and -145 that is associated with bone metastasis of prostate cancer and involved in the regulation of EMT. PLoS One. 2011;6:e20341.
44. Tavano F, di Mola FF, Piepoli A, Panza A, Copetti M, Burbaci FP, et al. Changes in miR-143 and miR-21 expression and clinicopathological correlations in pancreatic cancers. Pancreas. 2012;41:1280-4.
45. Achberger S, Aldrich W, Tubbs R, Crabb JW, Singh AD, Triozzi PL. Circulating immune cell and microRNA in patients with uveal melanoma developing metastatic disease. Mol Immunol. 2014;58:182-6.
46. Baffa R, Fassan M, Volinia S, O'Hara B, Liu CG, Palazzo JP, et al. MicroRNA expression profiling of human metastatic cancers identifies cancer gene targets. J Pathol. 2009;219:214-21.
47. Glud M, Rossing M, Hother C, Holst L, Hastrup N, Nielsen FC, et al. Downregulation of miR-125b in metastatic cutaneous malignant melanoma. Melanoma Res. 2010;20:479-84.
48. Liang L, Wong CM, Ying Q, Fan DN, Huang S, Ding J, et al. MicroRNA-125b suppressesed human liver cancer cell proliferation and metastasis by directly targeting oncogene LIN28B2. Hepatology. 2010;52:1731-40.
49. Rykov SV, Khodyrev DS, Pronina IV, Kazubskaya TP, Loginov VI, Braga EA. [Novel miRNA genes methylated in lung tumors]. Genetika. 2013;49:896-901.
50. Ueda T, Volinia S, Okumura H, Shimizu M, Taccioli C, Rossi S, et al. Relation between microRNA expression and progression and prognosis of gastric cancer: a microRNA expression analysis. Lancet Oncol. 2010;11:136-46.
51. Wang H, Tan G, Dong L, Cheng L, Li K, Wang Z, et al. Circulating MiR-125b as a marker predicting chemoresistance in breast cancer. PLoS One. 2012;7:e34210.
52. Cao J, Song Y, Bi N, Shen J, Liu W, Fan J, et al. DNA methylation-mediated repression of miR-886-3p predicts poor outcome of human small cell lung cancer. Cancer Res. 2013;73:3326-35.
53. Lin RJ, Xiao DW, Liao LD, Chen T, Xie ZF, Huang WZ, et al. MiR-142-3p as a potential prognostic biomarker for esophageal squamous cell carcinoma. J Surg Oncol. 2012;105:175-82.
54. Abraham D, Jackson N, Gundara JS, Zhao J, Gill AJ, Delbridge L, et al. MicroRNA profiling of sporadic and hereditary medullary thyroid cancer identifies predictors of nodal metastasis, prognosis, and potential therapeutic targets. Clin Cancer Res. 2011;17:4772-81.
55. Brase JC, Johannes M, Schlomm T, Falth M, Haese A, Steuber T, et al. Circulating miRNAs are correlated with tumor progression in prostate cancer. Int J Cancer. 2011;128:608-16.
56. Harris T, Jimenez L, Kawachi N, Fan JB, Chen J, Belbin T, et al. Low-level expression of miR-375 correlates with poor outcome and metastasis while altering the invasive properties of head and neck squamous cell carcinomas. Am J Pathol. 2012;180:917-28.
57. Hui AB, Bruce JP, Alajez NM, Shi W, Yue S, Perez-Ordonez B, et al. Significance of dysregulated metadherin and microRNA-375 in head and neck cancer. Clin Cancer Res. 2011;17:7539-50.
58. Kong KL, Kwong DL, Chan TH, Law SY, Chen L, Li Y, et al. MicroRNA-375 inhibits tumour growth and metastasis in oesophageal squamous cell carcinoma through repressing insulin-like growth factor 1 receptor. Gut. 2012;61:33-42.
59. Li J, Li X, Li Y, Yang H, Wang L, Qin Y, et al. Cell-specific detection of miR-375 downregulation for predicting the prognosis of esophageal squamous cell carcinoma by miRNA in situ hybridization. PLoS One. 2013;8:e53582.
60. Li Y, Jiang Q, Xia N, Yang H, Hu C. Decreased expression of microRNA-375 in nonsmall cell lung cancer and its clinical significance. J Int Med Res. 2012;40:1662-9.
61. Madhavan D, Zucknick M, Wallwiener M, Cuk K, Modugno C, Scharpff M, et al. Circulating miRNAs as surrogate markers for circulating tumor cells and prognostic markers in metastatic breast cancer. Clin Cancer Res. 2012;18:5972-82.
62. Wu X, Somlo G, Yu Y, Palomares MR, Li AX, Zhou W, et al. De novo sequencing of circulating miRNAs identifies novel markers predicting clinical outcome of locally advanced breast cancer. J Transl Med. 2012;10:42.

63. Yu H, Jiang L, Sun C, Li Guo L, Lin M, Huang J, et al. Decreased circulating miR-375: a potential biomarker for patients with non-small-cell lung cancer. Gene. 2014;534:60-5.
64. Wang L, Wang Q, Li HL, Han LY. Expression of MiR200a, miR93, metastasis-related gene RECK and MMP2/MMP9 in human cervical carcinoma--relationship with prognosis. Asian Pac J Cancer Prev. 2013;14:2113-8.
65. Xiao ZG, Deng ZS, Zhang YD, Zhang Y, Huang ZC. Clinical significance of microRNA-93 downregulation in human colon cancer. Eur J Gastroenterol Hepatol. 2013;25:296-301.
66. Zhang YK, Zhu WY, He JY, Chen DD, Huang YY, Le HB, et al. miRNAs expression profiling to distinguish lung squamous-cell carcinoma from adenocarcinoma subtypes. J Cancer Res Clin Oncol. 2012;138:1641-50.
67. Veerla S, Lindgren D, Kvist A, Frigyesi A, Staaf J, Persson H, et al. MiRNA expression in urothelial carcinomas: important roles of miR-10a, miR-222, miR-125b, miR-7 and miR-452 for tumor stage and metastasis, and frequent homozygous losses of miR-31. Int J Cancer. 2009;124:2236-42.

## SUPPLEMENTARY TABLE 2: The association between breast cancer patients overall survival and the Rho pathway

The association between TCGA BRCA patients overall survival (OS) with age, stage of disease and members of the Rho family (RHOA, RHOC, ROCK1, ROCK2, RHOBTB1, RHOBTB2, RHOBTB3, RHOB, RHOD, RHOF, RHOG, RHOH, RHOJ) and downstream ROCK-LIMK adhesion pathway (CFL1, CFL2, LIMK1, LIMK2, SSH1, SSH2, SSH3) was examined. Disease stage and 5 genes from the Rho-ROCK-LIMK family and pathway were associated ($p<0.05$) with OS of BRCA patients on univariate analysis. There is a high likelihood ($p<0.05$) that the 6-miRNA-signature score had interactive effects with 6 genes (RHOA, RHOBTB2, RHOC, RHOG, ROCK2, SSH3) from the cell adhesion pathway. From the 6 variables found to be associated with patient OS, 4 (RHOA, RHOBTB2, RHOC, RHOG) were highly likely to interact with the 6-miRNA-signature. Interaction analysis between BRCA tumor 6-miRNA-signature score with the expression of mRNAs from the Rho-ROCK-LIMK pathway, and estrogen receptor status was explored using Chi-Square tests.

| **Variables analyzed** | **Correlation with OS (p-value)** | **Probability of interaction with 6-miR score (p-value)** |
|---|---|---|
| Age | 0.08 | **0.009** |
| Stage | **<0.01** | 0.771 |
| ER status | 0.06 | **<0.001** |
| LIMK1 | 0.1 | 0.435 |
| RHOA | **0.03** | **0.021** |
| RHOBTB2 | **0.04** | **0.012** |
| RHOC | **0.05** | **<0.001** |
| RHOD | 0.08 | 0.287 |
| RHOG | **0.04** | **0.019** |
| ROCK2 | 0.08 | **0.007** |
| SSH1 | **0.05** | 0.330 |
| SSH3 | 0.07 | **<0.001** |
